# Prognostic Value of the Six-Minute Walk Test in Heart Failure Patients Undergoing Cardiac Surgery: A Literature Review

**DOI:** 10.1155/2013/965494

**Published:** 2013-07-24

**Authors:** Dominika Zielińska, Jerzy Bellwon, Andrzej Rynkiewicz, Mohamed Amr Elkady

**Affiliations:** ^1^Clinic of Rehabilitation, Medical University of Gdańsk, ul. Dębinki 7, 80-952 Gdańsk, Poland; ^2^Department of Cardiology, Medical University of Gdańsk, Poland; ^3^Department of Physical Medicine & Rehabilitation, Ain Shams University, Cairo, Egypt

## Abstract

*Background*. The prognostic value of cardiopulmonary exercise testing (CPET) is known, but the predictive value of 6MWT in patients with heart failure (HF) and patients undergoing coronary artery bypass grafting (CABG) is not established yet. *Objective*. We conducted a systematic review exploring the prognostic value of 6MWT in HF patients undergoing cardiac surgery. The aim was to find out whether the change in the distance walked during follow-up visits was associated with prognosis. *Data Source*. We searched “PubMed” from January 1990 to December 2012 for any review articles or experimental studies investigating the prognostic value of 6MWT in HF patients and patients undergoing cardiac surgery. *Results*. 53 studies were included in the review, and they explored the role of 6MWT in cardiology, cardiac surgery, and rehabilitation. The results did not show the relation between the six-minute walk distance and adverse events after CABG. The predictive power of the distance walked for death in HF patients undergoing cardiac surgery was not found. It is not yet proved if the change in the six-minute walk distance is associated with prognosis. The predictive power of the six-minute walk distance for death in HF patients undergoing cardiac surgery remains unclear.

## 1. Background

### 1.1. Definition and History of Implementation of the Six-Minute Walk Test

The six-minute walk test is a simple, inexpensive, and reproducible method for the assessment of exercise capacity. Implementation of the test does not require any advanced equipment or training for technicians. During the test, the patient walks the longest possible distance within the time of 6 minutes on the flat surface and can stop or slow down at any time and then resume walking during the test. The main result of the six-minute walk test (6MWT) is the distance covered by the patient in 6 minutes. The 6MWT shows good correlation with the peak VO2 from cardiopulmonary exercise test and is much easier to perform, and it reflects well the daily activities of the patients. However, many variables may influence this test, and, therefore, it should always be performed according to the strict given protocol. The 6MWT was proposed for the first time by Balke in 1963, and since the mid-1980s, it has been used more and more widely in different clinical conditions. This test is most commonly used in pulmonary diseases, but it has been successfully implemented also in patients with cardiovascular diseases, pre- and postsurgical treatment, different neurological disorders, and fibromyalgia or spinal muscular atrophy. However, it seems that the 6MWT is not so popular among cardiologists and cardiosurgeons as it should be.

### 1.2. Six-Minute Walk Test as a Tool for Assessment of Clinical Condition and Prognosis in Cardiovascular Diseases

Exercise capacity and tolerance are the most important factors in assessment of the clinical condition and prognosis of patients with cardiovascular and pulmonary diseases. Exercise capacity is the strong prognostic factor in heart failure patients and can be best described by cardiopulmonary exercise testing (CPET), mainly measuring the peak oxygen consumption. Unfortunately, such test requires complicated and expensive equipment, qualified technicians, experienced physicians, and frequent gas and volume calibration. All that makes CPET a complicated method reserved for specialized facilities.

 Several different walk tests have been described in effort to measure the functional capacity, including 2-minute walk test (2MWT), 6MWT, 12MWT, self-paced walk test, shuttle walk test, and 1-mile track walk. All these tests are inexpensive and relatively simple to perform. Among them, the 6MWT seems to be the most frequently used for clinical and research purposes. This test was proposed for the first time in cardiology by Balke [1] in 1963 and then by Guyatt et al. [2] in heart failure patients. The 6MWT distance (6MWTD) seems to correlate best with the maximal oxygen consumption. This test is easy to be administrated, inexpensive, well tolerated by patients, and reflects their daily activities.

The 6MWT does not measure the peak oxygen uptake or determine the cause of dyspnea on exertion, but it correlates well (*r* = 0,73) with VO2 peak in some patients with end-stage lung diseases.

Anyway, the 6MWT distance correlates better with the quality of life indices than with VO2 peak, and this demonstrates the 6MWT better suitability for assessing patients' ability in performing their daily activities than the CPX test [3].

In fact, there are many protocols of the 6MWT but the differences between them are usually small, but the most detailed and widely implemented protocol was published by the American Thoracic Society in 2002 [3].

The assessment of exercise capacity by means of the 6MWT is most frequently used in pulmonary and cardiac diseases. This test measures the distance a person can quickly walk on a flat, hard surface in the time of 6 minutes (the 6MWD). The 6MWT requires a 30-meter (100 ft) corridor, stopwatch, mechanical lap counter, two small cones to mark the turnover points, one chair that can be easily moved along the walking course to support the patient, worksheets on a clipboard, an available source of oxygen, a sphygmomanometer or other validated blood pressure measuring devices, a telephone, and a defibrillator. The length of the hallway should be marked every 3 meters with a cone, and the starting line should be marked on the floor using brightly colored tape. In case of repeating the test, it is important that it should be performed at the same time of the day and without any “warmup.” The patient should rest seated on a chair located near the starting line for at least 10 minutes before the test starts. Meanwhile, the contraindications for the test should be checked and identified; the pulse and blood pressure should be measured. Performing pulse oximetry is optional. The baseline and overall fatigue should be assessed using the Borg scale [4]. Before starting the test, the patient is instructed to walk as far as possible for 6 minutes, and during the walk test, only standardized phrases for encouragement must be used. After the test, the postwalk dyspnea and fatigue should be assessed by Borg scale, as well as the pulse and blood pressure. If the oximeter was used, the Sat O2 should be recorded, right after the test. The number of laps from the counter should be recorded and additional distance covered should be also marked with the use of the markers on the wall as distance guides to calculate the total distance of walk.

The normative data of the 6MWTD are based on several studies performed on healthy populations, and their results serve as a reference point for better understanding and proper interpretation of the 6MWT results. The 6MWT distance depends on anthropometric variables like age, gender, and weight. It also depends on the protocol specifications, mainly on verbal encouragement for the patient to continue the test and also on the results obtained by the patient in learning how to perform the test [5].

In the study of Gibbons et al. [6] in which younger people whose mean age was 45.1 years were examined and included, the participants achieved a mean 6MWT distance of 689 meters for men and 615 meters for women. 

Among other studies of more elderly people, Troosters et al. [7] reported a mean 6MWT distance of 613 meters in subjects whose mean age was 65 years, and the results reported were 656 meters for men and 554 meters for women. 

Steffen et al. reported a mean 6MWT distance of 505 meters for men and of 467 meters for women aged 74.1 on average [8]. 

Another study performed by Enrichi and Sherrill [9] provided normative data in healthy adults. They have examined 117 healthy men and 173 healthy women aged from 40 to 80 years, and the mean 6MWT distance was 576 meters for men and 494 meters for women. Based on those data, they developed equations allowing adjustment for gender, age, height, and weight to calculate the distance walked by a healthy adult during the 6MWT.

### 1.3. Six-Minute Walk Distance as a Prognostic Factor in Heart Failure Patients

Several studies have reported that the 6MWT is a reliable measure of increased mortality among cardiac patients, with the distance of less than 300 meters being a strong indicator of poor prognosis [10]. The 6MWT distance in patients with heart failure and left ventricular ejection fraction (LVEF) of 20% averages at 310 meters [11], whereas in those with mild disease and preserved LVEF (>53%), it is over 427 meters [12].

In patients with heart failure, a low 6-minute walk distance has been associated with increased total mortality and more hospital admissions for heart failure [13, 14]. The 6-minute walk distance is only weakly correlated with LV ejection fraction, and it provides independent prognostic information [13, 14].

The 6MWT is a safe and simple clinical method; that is, it strongly and independently allows us to predict heart failure hospitalization rates and mortality in patients with left ventricular dysfunction. The mortality was 3.5 times higher in subjects covering less than 350 meters in the 6MWT than in those who walked over 450 meters in the Studies of Left Ventricular Dysfunction (SOLVD) registry substudy [15]. Other studies support the usefulness of the 6MWT distance in predicting not only mortality but also hospitalization for heart failure [13, 16].

The 6MWT can differentiate the most severe heart failure patients from the ones with mild to moderate diseases. The 6MWD is inversely related to New York Heart Association (NYHA) functional class and quality of life (QoL). However, only the physical functioning sections of health-related quality of life questionnaires, like SF-36 or MLHFQ, correlate significantly with the 6MWT distance [17, 18]. In contrast, the nonphysical domains of quality of life do not correlate with the 6MWT.

Peak VO2 is a strong indicator of heart failure severity and is an important factor in timing of heart transplantation, and the 6MWT distance is strongly correlated with peak VO2 in HF patients with reported correlation coefficient in the range from *r* = 0.56 to *r* = 0.88 [5]. The correlation between 6MWT and peak VO2 in patients with heart failure is stronger in patients with low 6MWT and low peak VO2; then, 6MWT becomes less predictive as peak VO2 value becomes higher. The 6MWT is reliable, valid, and predictive for patients with heart failure who do not walk greater than 490 meters.

However, others have not confirmed this relationship, and they suggest that VO2 peak is a better predictor of survival, particularly over longer followup periods [19–22].

Some authors suggest that a submaximal exercise test could reflect the results obtained from a maximal exercise test in people whose physical functional capacity is severely impaired. However, maximal exercise testing may be more precise in those with severe heart failure who are referred for heart transplantation [3].

There are somehow conflicting results on using the 6MW distance as a marker of improvement following medical therapy for heart failure. In some cases, treatment with betablockers, angiotensin II blockers, or ACE inhibiters in general did not increase the 6MW distance despite the increase in LVEF and NYHA functional class [23–25]. 

An analysis by Olsson et al. [26] summarizing 39 studies related to the usefulness of the 6MWT as a measure of the medical treatment effectiveness presented only nine significant results. The majority of the pharmacological trials did not show any significant changes in the 6MWT distance. Nevertheless, a mean of 41 meters decrease in the 6MW distance has been observed by Packer et al. after digoxin withdrawal in patients with LVEF 35% in the RADIANCE Study [27]. Changes in 6MWT distance correlate better with changes in symptoms, and it may be used as supportive evidence for symptom benefit following different means of treatment in heart failure patients. Similarly, small but significant improvements in the 6MW distance have been documented by several studies after cardiac resynchronization device implantation [28–30] and during the treatment with continuous positive airway pressure [31].

The test seems to be also safe in patients with refractory heart failure during the preoperative period for heart transplant surgery [32]. Clearer and more robust documentation is available on improvement in functional exercise capacity measured by the 6MWT after cardiac rehabilitation [33–35]. The 6MWT is considered safe to assess the submaximal functional capacity and can be used as an alternative test to evaluate the results of interventions in patients with permanent cardiac pacemakers [36].

 Absolute contraindications for the test include a history of unstable angina or a heart attack during the previous month. Relative contraindications are resting tachycardia HR > 120 beats/min or uncontrolled arterial hypertension [37].

The test strongly and independently predicts morbidity and mortality in patients with heart failure, and it is more sensitive to deterioration than to improvement in heart failure symptoms [15].

### 1.4. Six-Minute Walk Test in Patients Undergoing Cardiac Surgery

Another field of application of walk test is postsurgical cardiac and pulmonary rehabilitation. In the literature, we can find reference values of the 6MWT in patients early after cardiac surgery [37, 38]. Some authors used the test in patients after lung volume reduction surgery in order to compare median sternotomy and thoracoscopic approaches. 

Slow walking speed is a component of frailty and is associated with poor muscle strength and reduced mobility. In recent studies, frailty was a significant independent predictor of mortality or need for institutional care after cardiac surgery [39]. In 133 elderly patients undergoing cardiac surgery, Afilalo et al. reported that slow gait speed defined as the time taken to walk 5 meters in >6 seconds was associated with a higher risk of in-hospital complications from surgery based on the STS criteria. This small study did not report associations with long-term outcomes [40, 41]. In patients referred for surgery for severe aortic stenosis, the six-minute walk distance predicted death, myocardial infarction, and stroke events independently from the EuroSCORE [42].

## 2. Conclusion

The relation between the six-minute walk distance and adverse events after CABG has not been evaluated. The predictive power of the six-minute walk distance for death in heart failure patients undergoing cardiac surgery was not assessed as well. The question: “is change in the six-minute walk distance during follow-up visits associated with prognosis in heart failure patients?” also remains unanswered.
